# Evaluation of a 3D-printed hands-on radius fracture model during teaching courses

**DOI:** 10.1007/s00068-023-02327-4

**Published:** 2023-07-31

**Authors:** Jonas Neijhoft, Jasmina Sterz, Miriam Rüsseler, Vanessa Britz, Lena Bepler, Verena Freund, Christian Horz, Dirk Henrich, Ingo Marzi, Maren Janko

**Affiliations:** 1 Department of Trauma-, Hand- and Reconstructive Surgery, Goethe University Frankfurt, Theodor-Stern-Kai 7, 60590 Frankfurt am Main, Germany; 2 Goethe University Frankfurt, Medical Faculty, Institute for Medical Education and Clinical Simulation, Frankfurt am Main, Germany

**Keywords:** Teaching, Training, Fracture models, Radius fractures, 3D printing, Traumatology

## Abstract

**Objective:**

This study aimed to evaluate the effectiveness of a 3D-printed hands-on radius fracture model for teaching courses. The model was designed to enhance understanding and knowledge of radius fractures among medical students during their clinical training.

**Methods:**

The 3D models of radius fractures were generated using CT scans and computer-aided design software. The models were then 3D printed using Fused-Filament-Fabrication (FFF) technology. A total of 170 undergraduate medical students participated in the study and were divided into three groups. Each group was assigned one of three learning aids: conventional X-ray, CT data, or a 3D-printed model. After learning about the fractures, students completed a questionnaire to assess their understanding of fracture mechanisms, ability to assign fractures to the AO classification, knowledge of surgical procedures, and perception of the teaching method as well as the influence of such courses on their interest in the specialty of trauma surgery. Additionally, students were tested on their ability to allocate postoperative X-ray images to the correct preoperative image or model and to classify them to the AO classification.

**Results:**

The 3D models were well received by the students, who rated them as at least equal or better than traditional methods such as X-ray and CT scans. Students felt that the 3D models improved their understanding of fracture mechanisms and their ability to explain surgical procedures. The results of the allocation test showed that the combination of the 3D model and X-ray yielded the highest accuracy in classifying fractures according to the AO classification system, although the results were not statistically significant.

**Conclusion:**

The 3D-printed hands-on radius fracture model proved to be an effective teaching tool for enhancing students' understanding of fracture anatomy. The combination of 3D models with the traditional imaging methods improved students' ability to classify fractures and allocate postoperative images correctly.

## Introduction

For decades, students were learning anatomy from books and drawings. While there exists visualization of basic and correct anatomy with the use of 3-dimensional (3D) models or teaching courses on human corpses, these techniques get rare in case of fracture models. But not only correct anatomy but also pathologies, fracture mechanisms, and basic understanding of surgical therapy are essential for the later clinical career of students. If commercial models are available, buying all of these is an expensive task. 3D-printing technologies like Fused-Filament-Fabrication (FFF) not only offer cost efficient, but innumerable replicability in production [1] of different fracture models which can be used for teaching purposes during courses for students [2].

In this study, a very common fracture should be addressed and aim to increase the understanding by demonstration and evaluation of 3D-printed models: Depending on country and population, fractures of the distal radius are the most common type with 10–25% of all fractures [3–5]. For that reason, the fracture of the distal radius is integrated with high skill levels within the German National Catalogue of Learning Objectives in Surgery (NKLC) for undergraduate medical students [6]. The fracture lines and mechanisms occur very homogeneous [7] and thus can be classified by the Arbeitsgemeinschaft für Osteosynthesefragen (AO) classification [8]. Primarily, they are grouped into A-, B- and C-fractures depending on their severity especially in terms of extra, partial, or complete intra-articular fracture lines.

Division in those classification groups is essential for choice of one of the different therapy options and has high impact on the outcome for the patients [9–11]. However, even consultants of trauma or orthopaedic surgery rarely seem to classify fractures the same, as multiple results of intra- and interobserver reliability studies show [12, 13]. Therefore, it is even more difficult for students and young residents to correctly assign fractures to the different classification systems. And since these fractures are so common, correct assignment followed by selection of the correct treatment options is even more important. 3D-printing concerns rather technical courses, but becomes more present in medical education, too. For education purposes themselves, it offers various advantages:

Studies by AlAli et al. (2018) have shown that the visualization of learning content in the form of 3D-printed illustrative models significantly increases the learning effect, for example with use in oral and cleft palate models in oral and maxillofacial surgery [14].

There are already commercially available models of many anatomy contents, but using 3D-printed models offers advantages in terms of cheap production and ad hoc availability of different norm variants or special fractures. Wang et al. (2017), for example, were able to show that no advantage could be achieved over a traditional model in comparison to the printed (single-colour) model of a heart [15]. Nevertheless, it would be advantageous to immediately show the learner the link between the diagnostic tools available in the clinical situation, such as X-rays and CT scans, and the anatomical relationship (of the fracture) on site, to possibly create a spatial understanding of the two-dimensional image data sets. Therefore, this study addresses the question of whether 3D-printed models can improve the teaching effectiveness and understanding of anatomy and fracture theory in medical students during their clinical training. In addition, the study will ask how students perceive this new teaching method and whether they think it is helpful.

## Methods

### Production of the models

3D printable models such as the described radius fractures in this study can be generated from daily CT scans or be manually designed through computer-aided-design. The whole procedure of generating the models is described in a previous study [2]. Shortly explained CT DICOM-data from our database were obtained and segmented into their bone and soft-tissue components using the software Slicer3D (*The Slicer Community*, Version 4.11). After limiting the bone to the region of interest, in the case of this study the distal radius and ulna, supports where added via Meshmixer (Version 3.5.474, *Autodesk*, San Rafael, USA). Before printing, the models need to be translated into a coordinate-based language, called GCODE, which is loaded onto the printer. For this process, Cura (Version 4.9, *Ultimaker*, Utrecht, The Netherlands) was used. After these steps, the models then were printed on a modified Ender 3 (*Creality,* Shenzhen, China). Parameters, which equal adequate details and printing speed, were used for printing. The most important selection of these is shown in Table 1. The printing times vary within the different models. In this study, 1–2 h per model were necessary.

**Table 1:** A selection of the printing parameters used to produce the models

Parameter	Value
Layer height	0.24 mm (for faster printing)
Infill density	10% (or above)
Top layers	3
Wall line count	3
Support overhang angle	80°

The parameters are varying depending on different printers

Adequate fractures then were segmented and 3D printed for each classification group. These models were then assessed as a blind sample by specialists and senior physicians.

### Ethics approval

The study was reviewed by the Ethical Commission of the University Hospital Frankfurt from Goethe University: It was stated that no ethical approval was required. Participation was voluntarily and the study was conducted according to the World Medical Association Declaration of Helsinki.

### Study population and questionnaire

**Fig. 1:**
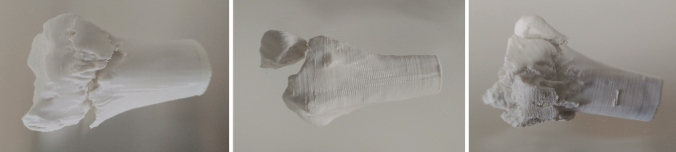
Finished and 3D-printed models of the three fracture types: left **A** fracture, middle **B**, and right **C** fracture

The study was performed as part of the obligatory training of practical skills in surgery for undergraduate medical students in their third of 6 years of education and after they had participated in the surgical lectures, which are based on the AO compendium [16]. The course and questionnaire were held in German language. Students participated voluntarily and after informed consent, which was revocable at any time. Participants first were assigned to three different groups. During the preparation course, fractures of the distal radius were explained to the students: For standardization, a video with relevant information about radius fractures was recorded and shown to the participants first. Then, each of the groups was handed out one of the three learning aids of fractures of the distal radius: conventional X-ray (R), CT data (CT), or a 3D-printed model (M) as shown in Fig. 1.

Afterwards, each student answered a short subjective questionnaire to evaluate the following questions:

- Did you understand fracture mechanism?
- Would you be able to correctly assign each fracture to the AO classification?
- Could you explain the surgical procedure?
- Could you explain the fracture to patients?
- Would you like to the get more teaching methods like this?
- Did the teaching method improve your interest in traumatology?

The evaluation was carried out using a six-point Likert scale from 1 “strongly agree” to 6 “strongly disagree”.

### Allocation test

**Fig. 2:**
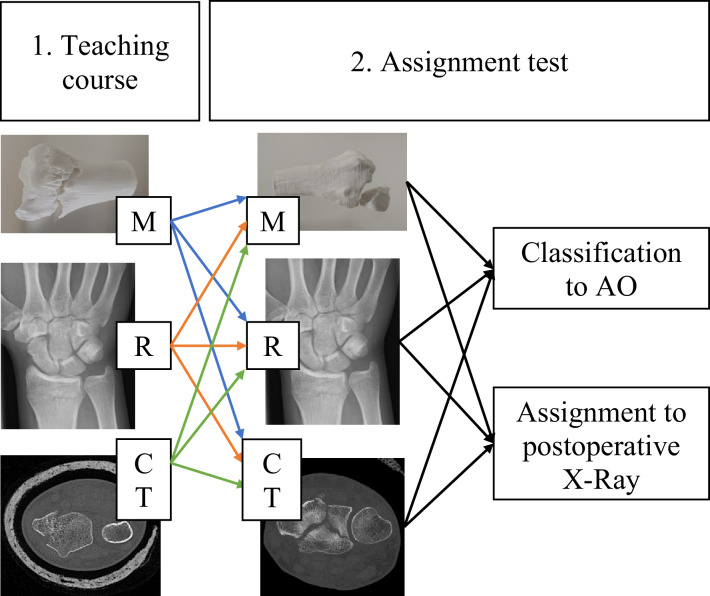
A schematic overview of the assignment test procedure is shown here. First, radius fractures were explained to the students during the course using a model (M), X-ray image (M), or CT (CT). This was followed by the assignment tests to AO or postoperative X-ray using a different model/X-ray/CT

After the first part, each group was handed out one other learning method or the same method with a different radius fracture. By this, a total of 9 (3^3^) groups were generated. Students had to assign these groups to the AO classification subgroup. In a last step, they got postoperative X-ray images, which also must be assigned to the right preoperative image or model, provided before. The test procedure is illustrated in Fig. 2.

### Statistical analysis

Statistical analysis was performed using SPSS (v.28.0.0.0, IBM, Armonk, USA) with sequential ANOVA tests and Tukey post hoc analysis.

## Results

**Fig. 3:**
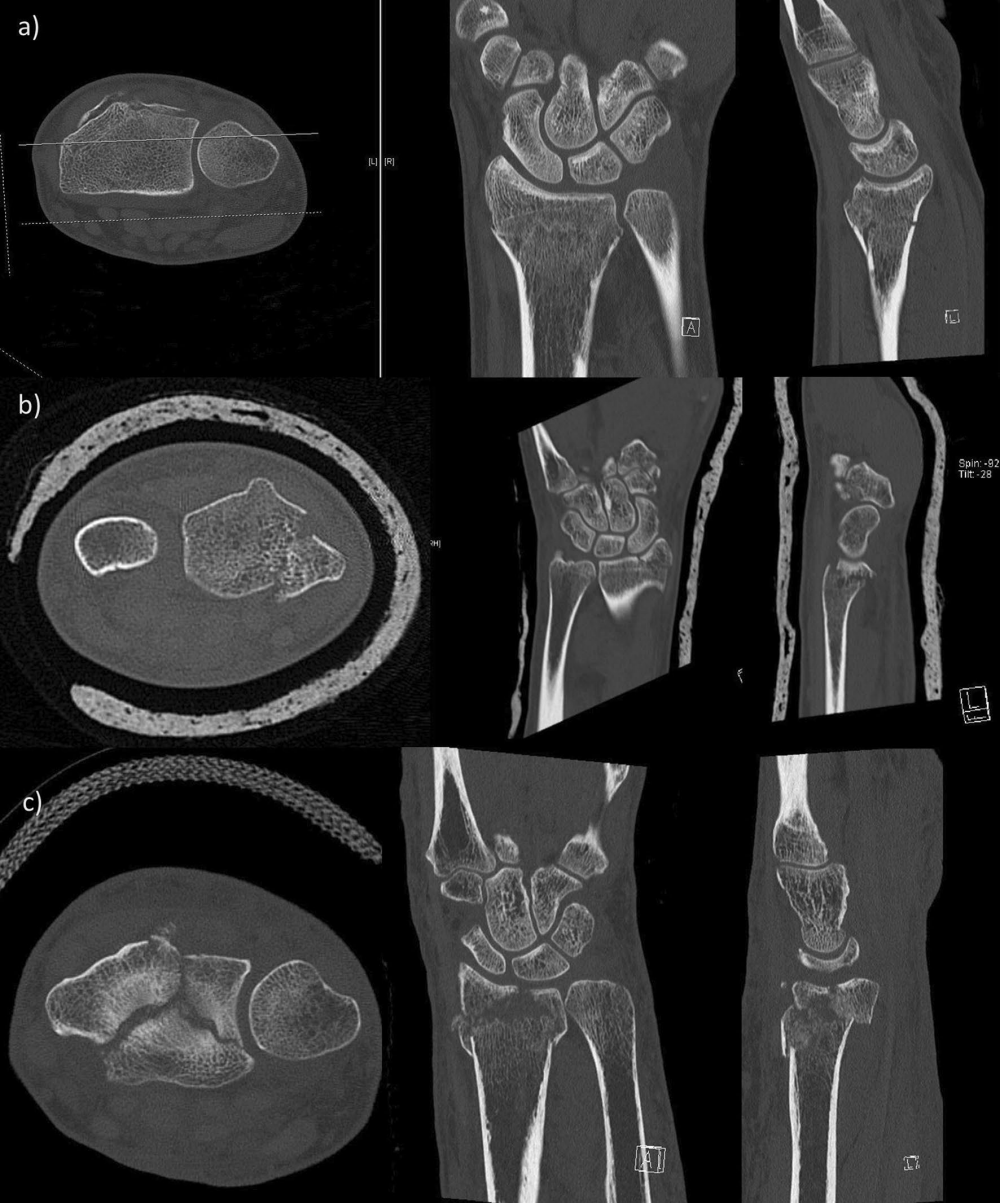
Example of CT scans used in the studies. Students were shown several slices and planes printed. **a** Distal radius fracture type a, **b** fracture AO b, and **c** fracture type c

**Fig. 4:**
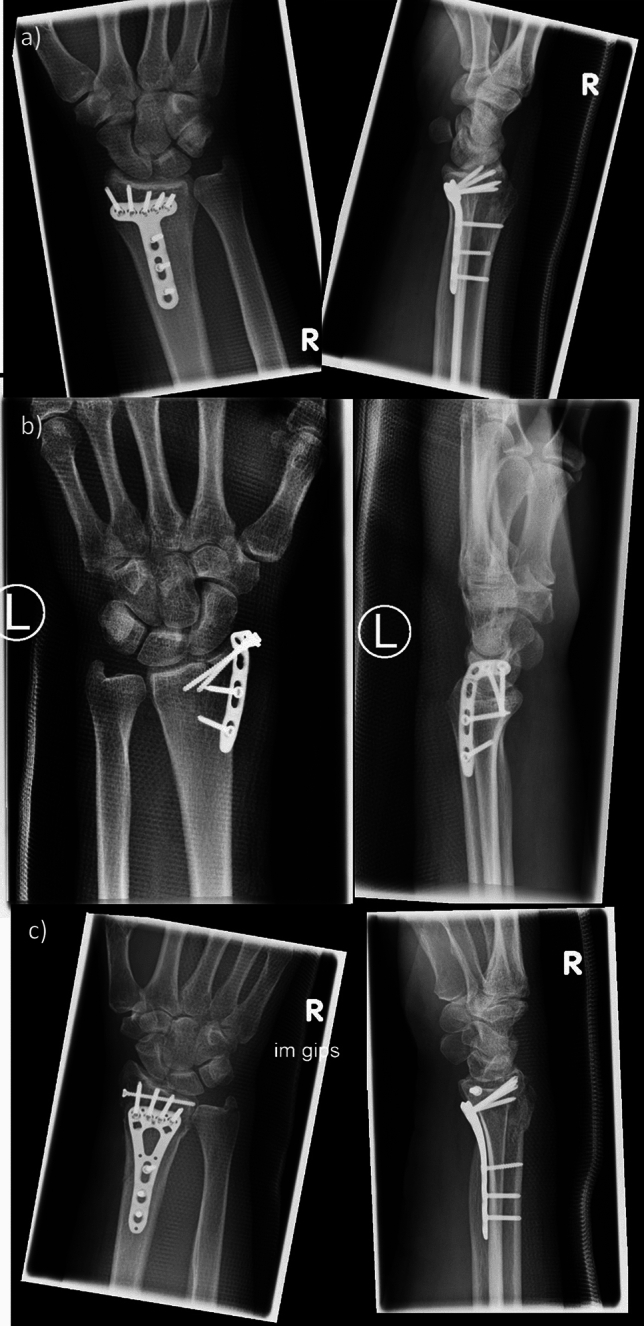
Postoperative surgery X-ray images. As there nearly exist no ct scans postoperatively, only X-ray images were shown and used for the assignment test. **a** Distal radius fracture type a, **b** fracture AO b, and **c** fracture type c

As previously described models were created and then printed. Each fracture model is shown in Fig. 1. Examples of each fracture are exemplary demonstrated as CT dataset in Fig. 3 and postoperative X-ray in Fig. 4.

### Evaluation of questionnaire

From the 170 participating students, 164 did completely answer the questionnaire, with 88 female and 76 male participants. Mean age was 24 (SD ± 3.6).

**Fig. 5:**
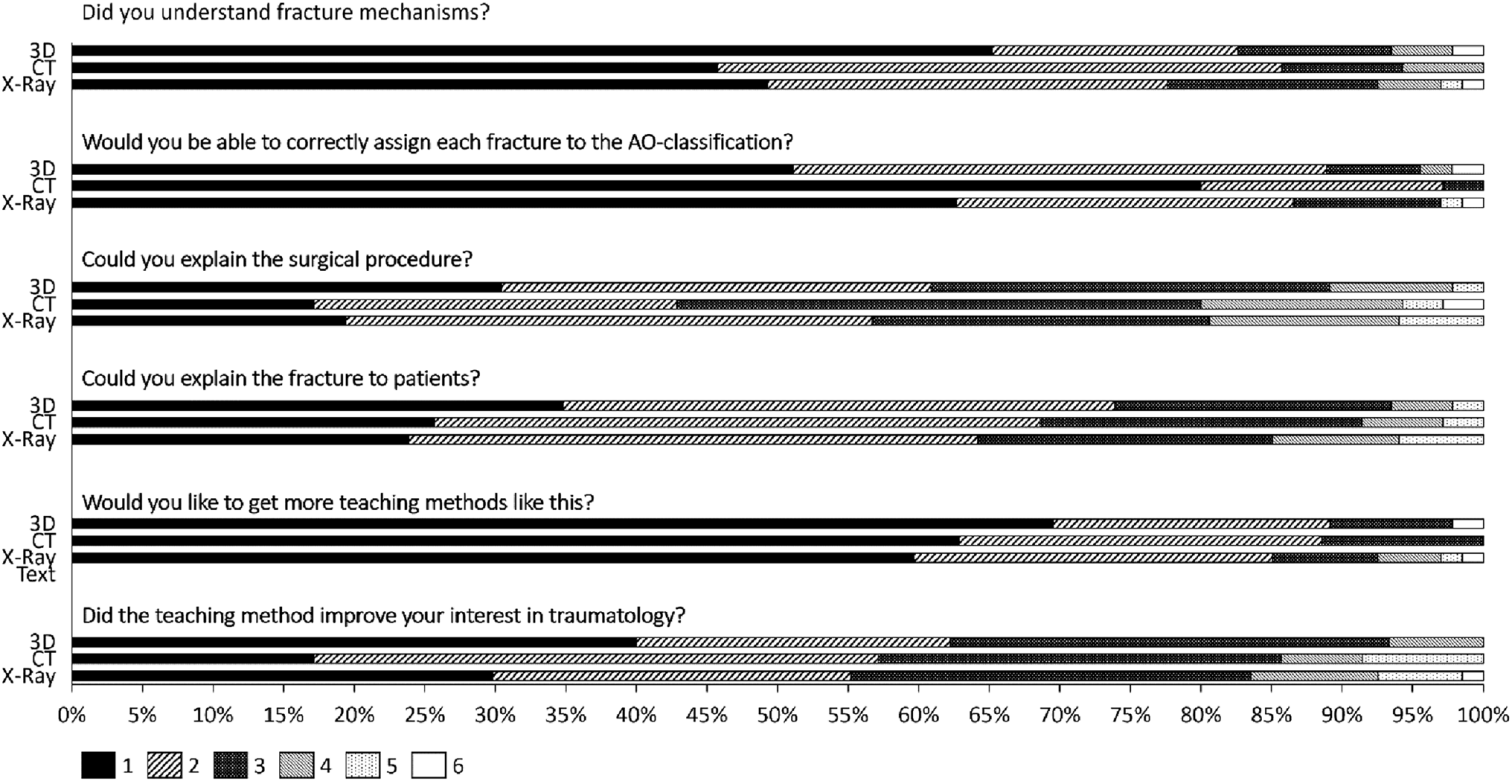
Graphical overview of the resulting quantity of answers from the Likert scale questionnaire: The answers range from 1 (“Strongly agree”) to 6 (“Strongly Disagree”)

Overall evaluation of the standard learning methods and the new models were widely accepted by the students, while the 3D models in each question were evaluated at least equally or better than the traditional methods like CT and X-ray. It is noticeable that students in self-assessment said that they understood fracture mechanisms for each learning type, with the 3D models being little ahead (M: Median = 1 CT: Median = 2 R: Median = 2). Also, students found themselves more capable of explaining the surgical procedure after they learnt from the 3D models (Median = 2), than from the CT scans (Median = 3), while the X-ray (Median = 2) was equal. The other answers to the questions did not differ from each other, while there are just small differences as seen in the interquartile ranges. In summary, students decided that courses with emphasis on fracture teaching are wished for the future (Median = 1 for all 3 groups) and did increase their interest on traumatology (Median = 2 for all 3 groups). A graphical overview over the results is given in Fig. 5.

### Allocation test results

The students were asked to allocate their fracture to the AO classification system, which yielded in the following dataset shown in Table 2. Results differ a lot, but the best outcome showed for the group which first got explanation by help of the 3D model and then were tested with the X-ray afterwards. Shortly behind was the explanation on CT scans, which then were tested with a 3D model second.

**Table 2:** Allocation of the fracture to AO classification system: M = 3D model, R = X-ray image, CT = CT scan, SD = standard deviation; for learning of the fractures each group was handed out the first model or image as encrypted with the first letter

Correct AO classification			
Group	Mean (%)	SD (%)	Group members
M_R	96.08	16	17
CT_M	95.24	17	14
R_CT	90.28	24	24
CT_CT	84.85	33	11
R_R	81.16	34	23
M_CT	75.64	38	26
R_M	75.00	36	20
M_M	71.79	43	13
CT_R	70.83	39	16

For the classification test, students got the learning method encrypted by the second letter, e.g., M_R learned with the 3D model (M) and were asked to classify a X-ray (R) image to the AO classification

Table 3 shows the results of the second test, where students had to assign their model or image to one postoperative X-ray image of the same fracture type. Best results were found for the combination of CT as learning and 3D models as testing technique, followed by the combination of the model for both steps. It also can be seen that test results were worst for combination of CT data images in both tests.

**Table 3:** Allocation of the fracture to postoperative X-ray images: M = 3D model, R = X-ray Image, CT = CT scan; for learning of the fractures each group was handed out the first model or image as encrypted with the first letter

Correct postoperative X-ray allocation			
Group	Mean (%)	SD (%)	Group members
CT_M	100.00	0	14
M_M	82.05	36	13
R_R	81.16	37	23
M_R	80.39	32	17
CT_R	79.17	37	16
R_CT	76.39	35	24
R_M	70.00	39	20
M_CT	69.23	43	26
CT_CT	63.64	41	11

For the allocation test procedure, students got the learning method encrypted by the second letter: for example, CT_M – students in this group learned with CT data (CT) during the course. For the test, they had to correctly assign a postoperative X-ray image to the corresponding 3D model (M)

## Discussion

Using FFF 3D printing for generating models for medical education is a promising teaching method with multiple advantages. However, 3D-printed models for teaching purposes do not only exist in trauma surgery, such as pelvic [17] or tibial fractures [18], but also in other specialties, e.g., oral and maxillofacial surgery [19, 20]. In this study, the outcome of teaching with different learning methods regarding distal radius fractures was investigated. The results show that the teaching method in fracture anatomy generally is rated with good marks and most of the students liked the course and saw advantages in it. The differences between each method (model, CT data, and X-ray) were small, but the reviews and responses regarding the model were even or better than the other methods. Interestingly, in the AO classification assignment test, the combinations showed the best test results, especially the combination of model/X-ray and CT/model. This might be based on different brain areas used for learning from plane 2D pictures or from holding a model in one´s own hand.

By addressing different brain areas, visual object recognition generates a plastic learning success. DiCarlo et al. (2012) are calling it “core object recognition”, which despite minor differences in the pattern (e.g., small variations of fracture lines in our cases), it allows the fracture to be recognized [21]. The problem of mentally transferring two- to three-dimensional images is also known in other disciplines, e.g., obstetrics with 2D ultrasound being changed to a 3D image, so that such tasks are increasingly being handed over to computer programs through deep learning. This is only possible to a limited extent in trauma surgery [22]. It is precisely this link between two-dimensional X-ray images or 3D-generated (but mostly 2D-displayed) CT data and the real examination findings as well as intraoperative situs that seems essential. Since the traditional teaching methods in the form of books and illustrations convey information primarily in two-dimensional form, the learner can only establish this link at an advanced stage in everyday clinical practice as soon as there are real points of contact, e.g., in the operating theatre. A large proportion of students do not experience parts of this experience during their education, although the surgical specialties in particular, which are finding it increasingly difficult to find young residents not only in Germany but also the rest of the world [23–25], could and thus should offer a great deal of plasticity. Taking this into consideration students mainly learn from books and digital media during their education, the good results in these groups in the tests could be explained by the experience in such learning media; nevertheless, it also shows that 3D models also generally perform well in the examination situation. Standard fractures are thus easily recognized via pattern recognition in X-rays or CT scans. As radiological images are the main diagnostic tools for fracture teaching in everyday trauma surgery, this is to be supported. However, it is not only the pure recognition of patterns that counts, but also—especially for medical explanation or surgical techniques—the understanding of the fracture mechanism and the proper therapy.

However, everyone learns differently. Such different learning types as visual, auditory, and haptic need to be addressed, and thus, it is of great advantage if different sensory impressions and methods are used in teaching. It is not surprising that it is precisely the combination of different teaching and learning methods that makes it much easier for students to understand and permanently remember the learning content [26].

Maybe, the low results of the inter- and intraobserver reliability of the results of classification systems like AO [27] can be improved, if the teaching of students already focuses on fracture teaching and its classification as part of the learning process. It could be further improved by 3D visualization of not only the fracture but also the classification system itself. In further studies, it might be an option to develop (3D printed) fracture models for the most common classification systems. With the advance of technology and widely available powerful computer systems, as well as the affordable maintenance and use of 3D printers, it is now possible to take this step of transferring and linking knowledge into the third dimension earlier. Furthermore, such learning concepts can be extended easily: for example, in addition to surgical approaches or anatomical landmark courses, the teaching of surgical techniques with real implementation, and use of Kirschner wires, plate- or screw osteosyntheses are also conceivable. By this, there could be points of contact for interested students earlier in their education and raise their interest in a surgical career.

The 3D models printed from PLA show no deformation or change during the course and were disinfected with a disinfectant after each run. Even autoclaving can be used for such models between courses [28]. By this, the models can be used several times and stored for long time without degradation [1].

Although a whole cohort of every student from 1 year was participating, one limitation is the single-center design of this study. However, with evaluation of further courses, not only multicenter studies as well as long-time retention of the learned contents are of particular interest. Another strength of the study is the possibility of increasing the interest of students in the field of trauma surgery and thus attracting young colleagues.

Taking everything into consideration, printing life-sized fracture models gives the advantage of holding and feeling the model with one´s own hand, instead of just looking at it. Taking the fracture fragments apart gives the opportunity to have a look from every angle and understand therapy options, surgical approaches, and screw placement. Afterwards, the fracture can be puzzled together again. Thus, such 3D models should be integrated into teaching of medical students.

## Data Availability

The datasets generated and analyzed during the current study are available from the corresponding author on reasonable request.
